# Supplementary material for: Bioinformatics prediction and experimental verification of key biomarkers for diabetic kidney disease based on transcriptome sequencing in mice
Source: PeerJ. 2022 Sep 20;10:e13932. doi: 10.7717/peerj.13932 (PMC9504448; doi:10.7717/peerj.13932)
Supplement: Table S1 [file peerj-10-13932-s001.docx]

Supplementary Table 1

|  | GSE86300 | GSE184836 |
| --- | --- | --- |
| Platform | GPL7546 | GPL21103 |
| Platform Name | Affymetrix GeneChip Mouse Genome 430 2.0 Array [CDF: Mm_ENTREZG_10] | Illumina HiSeq 4000 (Mus musculus) |
| Sample information | 5 BKS db/m mice and 5 db/db mice  24 weeks old | 3 renal glomeruli from kidneys of db/db and db/m mice;  3 renal tubules from kidneys of db/db and db/m mice;  24 weeks old |
