# Supplementary material for: Bioinformatics prediction and experimental verification of key biomarkers for diabetic kidney disease based on transcriptome sequencing in mice
Source: PeerJ. 2022 Sep 20;10:e13932. doi: 10.7717/peerj.13932 (PMC9504448; doi:10.7717/peerj.13932)
Supplement: Table S2 [file peerj-10-13932-s002.docx]

Supplementary Table 2

| Primer | Sequence (5’-3’) | |
| --- | --- | --- |
| mus-Aacs-F | CCAGCACCTGAAGGAGCACATG |  |
| mus-Aacs-R | GACACCATCCAGTTCCACATCATCC |  |
| mus-β-actin-F | CTACCTCATGAAGATCCTGACC |  |
| mus-β-actin-R | CACAGCTTCTCTTTGATGTCAC |  |
| mus-Abcc4-F | CGGATCGGCACCATGACACTTC |  |
| mus-Abcc4-R | AAGGATTTCACCAACGGCTCACAG |  |
| mus-Fmo5-F | CGTGTTCGGAAGCCTCTGATGAC |  |
| mus-Fmo5-R | AGCCAGGATGACAGCAAAGAAAGC |  |
| mus-Apoh-F | GCAGAGATGGCACTATCGAGATTCC |  |
| mus-Apoh-R | CGACTTCAGCACGGTGTCAGTTC |  |
| mus-Akr1c14-F | TGCACATTGGGAAGTTCACGAGAC |  |
| mus-Akr1c14-R | CACGCTGTAATTGGTAACGAATGGC |  |
| mus-Lpl-F | CGCTCTCAGATGCCCTACAAAGTG |  |
| mus-Lpl-R | TTGTGTTGCTTGCCATCCTCAGTC |  |
| mus-Cpe-F | CCGAGACCAAGGCTGTCATTCAC |  |
| mus-Cpe-R | CGTCATCAGGGCAGGAACTGTATTC |  |
| mus-Kynu-F | GTCTTGCATTTCACCACGCTTTCTC |  |
| mus-Kynu-R | ACGCTGAGTGCTAAGTTGATGTCTG |  |
| mus-Cyp7b1-F | GCCTCTCTAGCAAACACCATTCCAG |  |
| mus-Cyp7b1-R | AATTTCGTCACGCAGGGCTTCC |  |
| mus-Gsta2-F | CCCAGACCAAAGAGAAGCCAAGAC |  |
| mus-Gsta2-R | GCCTGTTGCCCACAAGGTAGTC |  |
| mus-Slc1a4-F | GGAAGACACCACCACAACTGACTG |  |
| mus-Slc1a4-R | CAGGCTTGATGGCTGTGCTACG |  |
| mus-Cd36-F | TGATACTATGCCCGCCTCTCCTG |  |
| mus-Cd36-R | CATCGTTTCCCACACTCCTTTCTCC |  |
| mus-Slc22a7-F | GAAGCAGCCTTTACTACAGCCTACC |  |
| mus-Slc22a7-R | GCCACACTCCATCCAGCAAGAC |  |
