# Supplementary material for: Bioinformatics prediction and experimental verification of key biomarkers for diabetic kidney disease based on transcriptome sequencing in mice
Source: PeerJ. 2022 Sep 20;10:e13932. doi: 10.7717/peerj.13932 (PMC9504448; doi:10.7717/peerj.13932)
Supplement: File S2 [file peerj-10-13932-s005.pdf]

```
rm(list = ls())#remove
exp <- read.csv('file.csv')

# Use the Avereps function of the Limma package to remove the repetition
library(limma)
symbol_unique <- avereps(exp[,-c(1)],
                         ID=exp$gene_name)
table(duplicated(row.names(symbol_unique)))
write.csv(symbol_unique,'file.csv')

inputFile="geneMatrix.txt"          # Input file
conFile="sample1.txt"               # Control sample
treatFile="sample2.txt"             # Experimental sample
logFCfilter=1                       #logFC
adj.P.Val.Filter=0.05               # P. value
geoID="data"                        #GEO database id

setwd("C:/Users/Desktop/group")     # Setting the Working Directory
getwd()
# Read the input file and organize the input file
rt=read.table(inputFile,sep="\t",header=T,check.names=F)
rt=as.matrix(rt)
rownames(rt)=rt[,1]
exp=rt[,2:ncol(rt)]
dimnames=list(rownames(exp),colnames(exp))
data=matrix(as.numeric(as.matrix(exp)),nrow=nrow(exp),dimnames=dimnames)
data=avereps(data)
data=data[rowMeans(data)>0,]
data=log2(data+1)          # If the representation is large, we need to take log2 of the data.
data=normalizeBetweenArrays(data)

# Read sample information
sample1=read.table(conFile,sep="\t",header=F,check.names=F)
sample2=read.table(treatFile,sep="\t",header=F,check.names=F)
sampleName1=gsub("^ | $", "", as.vector(sample1[,1]))
sampleName2=gsub("^ | $", "", as.vector(sample2[,1]))
conData=data[,sampleName1]
treatData=data[,sampleName2]
data=cbind(conData,treatData)
conNum=ncol(conData)
treatNum=ncol(treatData)

# Genetic difference analysis
Type=c(rep("con",conNum),rep("treat",treatNum))
```

```
design <- model.matrix(~0+factor(Type))
colnames(design) <- c("con","treat")
fit <- lmFit(data,design)
cont.matrix<-makeContrasts(treat-con,levels=design)
fit2 <- contrasts.fit(fit, cont.matrix)
fit2 <- eBayes(fit2)

allDiff=topTable(fit2,adjust='fdr',number=200000)
allDiffOut=rbind(id=colnames(allDiff),allDiff)
write.table(allDiffOut,file=paste0(geoID,".all.txt"),sep="\t",quote=F,col.names=F)

# Output the corrected expression amount
outData=rbind(id=paste0(colnames(data),"_",Type),data)
write.table(outData,file=paste0(geoID,".normalize.txt"),sep="\t",quote=F,col.names=F)

# Output difference result
diffSig=allDiff[with(allDiff, (abs(logFC)>logFCfilter & adj.P.Val < adj.P.Val.Filter )), ]
diffSigOut=rbind(id=colnames(diffSig),diffSig)
write.table(diffSigOut,file=paste0(geoID,".diff.txt"),sep="\t",quote=F,col.names=F)
```
